# Esophagogastroduodenoscopy in Patients With Dyspepsia: A Retrospective Study at a Tertiary Hospital

**DOI:** 10.7759/cureus.36520

**Published:** 2023-03-22

**Authors:** Hussain Yousif Alamen Abdalla, Nassir Alhaboob Arabi, Abdelmaged Mohammed Musaad, Anas E Elsheikh, Nasser Alrashidi

**Affiliations:** 1 Surgery, Faculty of Medicine, Soba University Hospital, Khartoum, SDN; 2 Surgery, Unaizah College of Medicine and Medical Sciences, Qassim University, Al-Qassim, SAU

**Keywords:** (nsaid) non-steroidal anti-inflammatory drugs, esophagogastroduodenoscopy (egd), sudanese patients, symptomatology, dyspepsia

## Abstract

Background

Recurrent upper abdominal pain or dyspepsia is one of the patients' most common chief complaints. This study correlates the symptoms of dyspepsia to esophagogastroduodenoscopy findings among Sudanese patients attending Soba University Hospital.

Methods

A retrospective observational study was conducted at Soba University Hospital from April 2019 to April 2020. Patients were selected according to inclusion and exclusion criteria. Each patient filled out a standardized data collection form with data on their demographics, symptoms, and endoscopic findings. A* P-value* of < 0.05 was considered statistically significant.

Results

The study included 142 patients, where 57.7% (n=82) were females, and 59.9% (n=85) of the study participants were of normal body mass index. In contrast, 57% of the study participants had a symptom duration of less than six months. Approximately 95.1% (n=135) suffered from heartburn, 80.3% (n=114) suffered from epigastric fullness, and 96.5% (n=137) suffered from epigastric pain. Seventy-eight point two percent (78.2%; n=111) who suffered from epigastric pain mentioned that it increased in intensity with food, 85.9% (n=121) who suffered from epigastric pain mentioned that it decreased in intensity with food, 54.2% (n=76) of the study participants suffered from regurgitation, 59.9% (n=85) of the study participants suffered from weight loss, 52.1% (n=73) of the study participants were using non-steroidal anti-inflammatory drugs (NSAIDs), 41.7% (n=59) of the study participants had normal esophagogastroduodenoscopy findings, followed by 35.9% (n=51) who had duodenitis or gastritis during the endoscopic assessment.

Conclusion

The study showed that esophagogastroduodenoscopy is not recommended in young patients without alarm symptoms who can be managed conservatively. However, every patient with alarming symptoms should have an esophagogastroduodenoscopy. Also, the study revealed that females and old-aged patients had higher rates of dyspeptic symptoms.

## Introduction

Dyspepsia is one of the most common reasons people seek medical care. It is characterized by the presence of heartburn and/or abdominal pain or discomfort in the upper central abdomen [1,2]. When considered individually, the clinical importance of upper abdominal symptoms is low, but it is more obvious when considered collectively. Various studies have suggested that psychosocial factors may be responsible for the symptoms of non-ulcer dyspepsia; however, it is still debatable whether symptoms can be used to accurately predict a diagnosis of functional dyspepsia instead of chronic peptic ulceration or other organic diseases [3,4].

In the general population, 25-40% of people report having symptoms of chronic upper abdominal pain or dyspepsia. More than 11 million clinician consultations were accounted for by these symptoms each year [5,6]. Therapeutic trials, Helicobacter pylori testing, upper gastrointestinal radiography, and esophagogastroduodenoscopy (EGD) are just a few diagnostic procedures that can assess dyspepsia [7-9]. Due in large part to the various evaluations of these models' accuracy, computerized decision analysis research has produced conflicting results about the best diagnostic and treatment approach [10-12]. According to a randomized controlled clinical trial, guided therapy administered after the initial EGD is associated with fewer expenses and fewer missed workdays than empiric therapy with histamine type-2 receptor antagonists (H2 blockers) [13].

Most dyspepsia-related disorders, such as peptic ulcer disease, gastric cancer, gastroduodenitis, and esophagitis, are best diagnosed via EGD. However, some physical discomfort, sizable social inconvenience, and expenses are associated with EGD [14]. The success of efforts to pinpoint the patients most likely to gain from endoscopy has been limited. It has been demonstrated that clinical indicators, such as dyspepsia subtypes (ulcer-like, reflux-like, and motility-like), do not accurately predict pathological diseases. Age and "alarm symptoms," including weight loss, repeated vomiting, dysphagia, bleeding, or anemia, are additional characteristics that can be predictive in some studies but not in others [15]. According to the American Gastroenterological Association, all patients above the age of 45 and those with alarm symptoms should get an EGD. Over a six-month period, dyspepsia affects 30-40% of adults, resulting in 2-3% of primary care consultations and about 40% of referrals to gastroenterologists. Therefore, patients with more severe symptoms or those for whom treatment has failed are more likely to be chosen for EGD than patients with less severe symptoms [16]. Gastroesophageal reflux disease (GERD), which has become more common although peptic ulcer disease and Helicobacter pylori infection have declined, was previously shown to be accurately predicted by endoscopies in roughly two-thirds of patients [17,18]. As a result, this study aims to correlate dyspepsia symptoms with EGD results in Sudanese patients to identify high-risk groups that require endoscopic interventions to minimize unnecessary EGD assessments.

## Materials and methods

This cross-sectional, descriptive hospital-based study was conducted at Soba University Hospital, which has a high-volume center for EGD with experienced endoscopists. The study was conducted during the period from April 2019 to April 2020. We included all patients with dyspepsia and planned for elective EGD in the study. In addition, we included both genders and those who gave consent to be part of the study. We excluded those dyspeptic patients with upper GI bleeding, those with a known diagnosis of previous EGD, those who underwent esophageal or gastric surgery, pregnant women with dyspeptic symptoms, and those aged less than 15 years. For data collection, the clerking sheet was filled out by a physician. Data regarding their presenting symptoms and signs (i.e., perianal pain, discharge, itching, swelling, abdominal pain, diarrhea, constipation, tenesmus, weight loss, and anorexia), bedside examination, endoscopy results, and histopathology investigation were also recorded. In addition, patients' demographic data (i.e., age and gender), as well as the clinical (i.e., rectal bleeding amount, duration, and color) and histopathological (i.e., location, the distance of the tumor from the anus and histology) features, were collected.

Microsoft Excel (Microsoft Corporation, Redmond, WA) recorded all collected responses accurately. The responses were then coded and imported into the IBM Statistical Package for Social Sciences, Version 25 (IBM Corp., Armonk, NY) for data analysis. An impartial biostatistician conducted the data analysis. Categorical data were represented in descriptive statistics in the form of frequencies and percentages utilizing appropriate tables and figures. The association between categorical variables was tested using Pearson's chi-square, and a *P-value* of less than 0.05 was considered statistically significant.

## Results

We found that 42.3% (n=60) of the study participants were in the age group (30-50) years old, with a mean age of (42.1 years SD ± 10). Furthermore, the majority were female 57.7% (n=82), with a female: male ratio of 1.4:1.

Seventy-eight point nine percent (78.9%; n=112) of the participants came from the Khartoum state geographical zone of Sudan, and 10.6% (n=15) were from the central geographical zone of Sudan. Of most study participants, 59.9% (n=85) had an average body mass index. In addition, 57% (n=80) had symptoms for less than six months.

**Figure 1:**
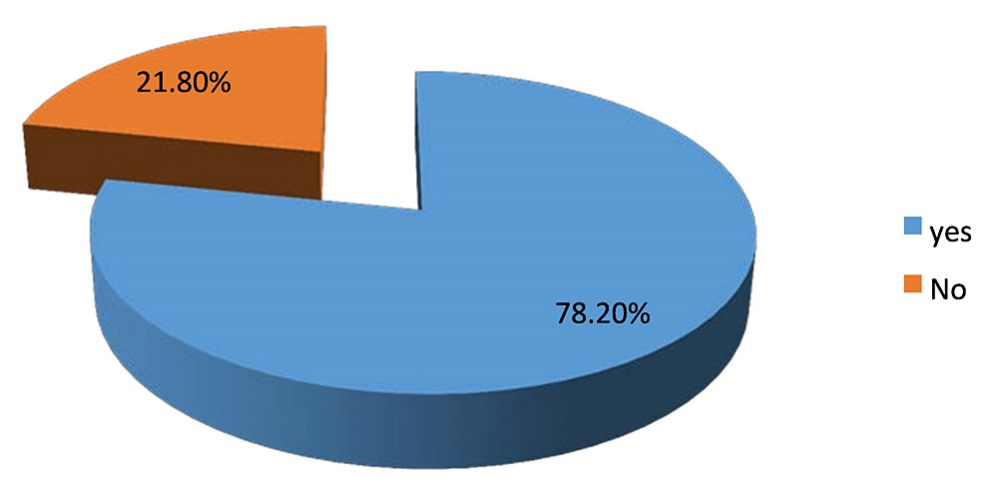
Epigastric pain increased intensity with food distribution among study participants, N=142

**Figure 2:**
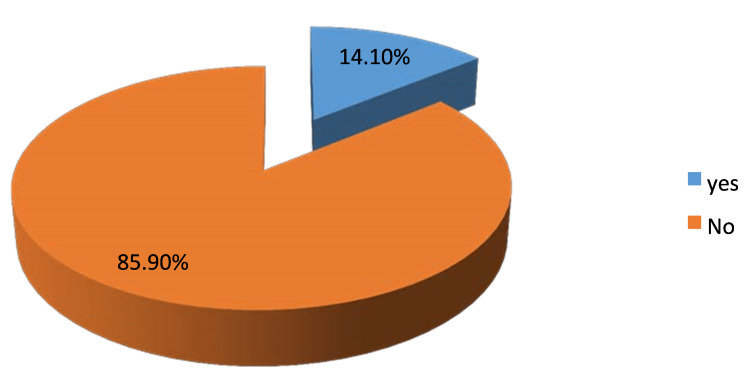
Epigastric pain decreased intensity with food distribution among study participants, N=142

Concerning the clinical presentations, our study found that 95.1% (n=135) of the participants suffered from heartburn, whereas 80.3% (n=114) of them presented with epigastric fullness. Furthermore, among the participants, 96.5% (n=137) of them had epigastric pain, of which 78.2% (n=111) increased intensity with food, 14.1% (n=121) decreased intensity with food; see Figure 1 and Figure 2, respectively. On the other hand, 54.2% (n=76) of the study participants suffered from regurgitation. Regarding dyspepsia's risk factor, our study found that 52.1% (n=73) of the participants used non-steroidal anti-inflammatory drugs (NSAIDs) (see Table 1).

**Table 1:** Demographic and symptoms among study participants, N=142 NSAID: non-steroidal anti-inflammatory drug

Variables	Frequency	Percent (%)
Age ( years)		
<30	27	19.7
30-50	60	42.3
>50	55	38
Gender		
Male	60	42.3
Female	82	57.7
Residence		
Northern	1	0.7
Central	15	10.6
Khartoum	112	78.9
Eastern	2	1.4
Western	12	8.4
Body mass index (kg/m^2^)		
<18.5	25	16.9
18.5-24.9	85	59.9
25.0 -29.9	30	21.1
>or=30	2	2.1
Duration of symptoms (months)		
<6	80	57
6-12	57	40.2
>12	5	2.8
Symptoms		
Heartburn	112	78.9
Epigastric fullness	114	80.3
Epigastric pain	137	96.5
Regurgitation	76	54.2
NSAID usage	73	52.1

Concerning the endoscopic findings, the study found that 41.7% (n=59) had normal endoscopic findings, while others showed abnormal endoscopic findings, as detailed in Table 2. In this study, cross-tabulation was done between risk factors (obesity, NSAIDs), age group, and duration of symptoms, in correlation with endoscopic findings. We found that there is a statistically significant correlation between endoscopic findings and age group between 30 and 50 years old where we commonly found normal endoscopic findings, while the age group of more than 50 years old was found with duodenitis and gastritis (*P-value* 0.02 ). Additionally, there is a statistically significant correlation between the duration of the symptoms and the endoscopic findings. In cases where the symptoms persisted for less than 12 months, we found duodenitis, gastritis, or normal endoscopic findings more frequently. In cases where the symptoms persisted for more than 12 months, we found gastrointestinal stromal tumors (GISTs) and polyps (*P-value* 0.001). Regarding the risk factor, there is a statistically significant difference between using NSAIDs and endoscopic findings, most commonly, duodenitis, gastritis, and peptic ulcer (*P-value* 0.01 ), as shown in Table 3.

**Table 2:** Endoscopic findings distribution among study participants, N=142 GERD: gastroesophageal reflux disease; GIST: gastrointestinal stromal tumor

Endoscopic findings	Frequency	Percentage (%)
Normal	59	41.7
Duodenitis-gastritis	51	35.9
Ulcer	10	7
Esophagitis	7	4.9
Hiatus hernia	5	3.5
GERD	4	2.8
GIST	4	2.8
Polyp	2	1.4
Total	142	100

**Table 3:** Variables and endoscopic findings cross-tabulation distribution among study participants, N=142 NSAID: non-steroidal anti-inflammatory drug

Variables	Intervals	Normal	Duodenitis gastritis	Ulcer	Esophagitis	Hiatus- hernia	GERD	GIST	Polyp	P-value
Age	<30	14	7	3	2	1	1	0	0	0.02
	30-50	29	16	5	5	2	1	1	1	
	>50	16	28	2	0	2	2	3	1	
Gender	Male	37	29	4	5	3	1	2	1	0.11
	Female	22	22	6	2	2	3	2	1	
BMI	<18.5	9	7	2	1	1	2	1	1	0.14
	18.5-24.9	36	35	4	4	3	0	2	1	
	25-29.9	14	8	3	2	1	1	1	0	
	>=30	0	1	1	0	0	1	0	0	
Symptoms duration	<6m	43	24	5	2	2	2	0	0	< 0.001
	6-12m	16	26	5	5	3	2	2	1	
	>12m	0	1	0	0	0	0	2	1	
NSAIDs usage	Yes	27	31	7	2	2	2	2	1	0.01
	No	32	20	3	5	3	2	2	1	

## Discussion

In our study, less than half (42.3%) of the study participants were in the age group 30-50 years old with a mean age of 42.1 ± 2 years, in a similar context to GNJ Tytgat et al. They reported that the mean age group of study participants was 45±2.4 years, with an increase in the mean age increasing the organic causes of dyspepsia, esophagitis, gastric ulcer, gastric tumor, and duodenal tumor [19]. Moreover, Wallace MB et al. reported that the mean age of study participants was 47.3 ± 1.6 years [20].

In our study, more than half (57.7%) of the study participants were female. However, there was no difference in endoscopic findings in both genders, in agreement with Wallace MB et al., who revealed that the dominant gender among their study participants was females 52% [20]. In addition, the Bashar Akram et al. study showed that 60.83% of patients were female and 39.1% were male [4].

In this study, we documented that 92.3% of the study participants were normal weight, followed by 5.6% complaining of weight loss, and 57% of the study participants had symptoms duration of fewer than six months. In a similar context with GNJ Tytgat et al. documented that the major complaints among their study participants were weight loss [19].

In our study, 95.1% suffered from heartburn, 80.3% suffered from epigastric fullness, 96.5% suffered from epigastric pain, 78.2% who suffered from epigastric pain mentioned that it increased with food, nearly 85.9% who suffered from epigastric pain said that it decreases with food, 54.2% of the study participants suffered from regurgitation, and 59.9% of the study participants suffered from weight loss. In the Ahmed Babi et al. study, epigastric pain was the predominant symptom at 79.2%, followed by heartburn at 26.1%. In agreement with GNJ Tytgat et al., who revealed that most of the study participants had suffered from epigastric pain and heartburn, 89.4% and 93.5%, respectively [19].

This study revealed that 52.1% of participants were using non-steroidal anti-inflammatory drugs. It is similar to Wallace MB et al., which documented that around 40% of their study participants were consuming NSAIDs [20], in addition, Nesland A et al. reported that more than 80% of study participants consumed NSAIDs [21].

In our study, less than half (41.7%) of the study participants had normal endoscopic findings, followed by more than a third (35.9%), who had duodenitis or gastritis. In a similar context to Bashar, Akram et al. concluded that a normal endoscopic finding was found in 40% of dyspeptic patients, followed by a duodenal ulcer in 18.33% [4]. And Wallace MB et al. reported that the main endoscopic findings among study participants were gastritis [20]. The Lamia AA et al. study results showed 73.2% non-erosive gastritis followed by 70.9% small hiatus hernia [22], in contrast to Nesland A et al., who reported dominant endoscopic findings as ulceration [21]. A significant statistical association was found between age and endoscopic findings; in those aged more than 50 years, their findings were gastritis, duodenitis, and GIST, in a similar context with Wallace MB et al. who documented a correlation between ages more than 45 years, and endoscopic findings as tumor and ulcer [20]. A significant statistical association was found between the duration of symptoms, more than 12 months, and endoscopic findings, as GIST, in a similar context with Maurizio R et al., who documented that a presentation of more than 12 months is significant gastric neoplasia and Barrett's esophagus [23].

A significant statistical association was found between NSAID consumption and endoscopic findings, such as gastritis and duodenitis, in a similar context to Nesland A et al. revealed statistical association between the frequency of NSAID consumption and endoscopic findings such as gastric and duodenal ulcers [21].

Limitations of the study

Our study was influenced by limitations related to its retrospective study design and secondary data sources. Consequently, essential variables that couldn’t be recorded in patients’ medical records and the limited number of cases due to the circumstances of the COVID-19 pandemic.

## Conclusions

According to the study, esophagogastroduodenoscopy is unnecessary in young people who don't have alarming symptoms because they can be managed without endoscopic intervention. However, esophagogastroduodenoscopy is strongly advised for all patients who have alarm symptoms. In females, dyspepsia symptoms occur more frequently and more in the old-age group of patients. In addition, non-steroidal anti-inflammatory drug users are more likely to have positive endoscopic findings.
